# Polymorphisms in mitochondrial ribosomal protein S5 (*MRPS5*) are associated with leprosy risk in Chinese

**DOI:** 10.1371/journal.pntd.0008883

**Published:** 2020-12-23

**Authors:** Yan Xing, Jun He, Yan Wen, Jian Liu, Yuangang You, Xiaoman Weng, Lianchao Yuan, Li Xiong, Xiaohua Chen, Ying Zhang, Huan-Ying Li

**Affiliations:** 1 Beijing Tropical Medicine Research Institute, Beijing Friendship Hospital, Capital Medical University, Beijing, China; 2 Beijing Key Laboratory for Research on Prevention and Treatment of Tropical Diseases, Beijing, China; 3 Leprosy Research Center, Yunnan Center for Disease Control and Prevention, Kunming, China; 4 State Key Laboratory for the Diagnosis and Treatment of Infectious Diseases, National Clinical Research Center for Infectious Diseases, The First Affiliated Hospital, Zhejiang University School of Medicine, Hangzhou, China; Shandong Provincial Institute of Dermatology and Venereology, CHINA

## Abstract

Leprosy is an infectious disease caused by *Mycobacterium leprae* (*M*. *leprae*), with about 210,000 new cases per year worldwide. Although numerous risk loci have been uncovered by genome-wide association studies, the effects of common genetic variants are relatively modest. To identify possible new genetic locus involved in susceptibility to leprosy, whole exome sequencing was performed for 28 subjects including 14 patients and 12 unaffected members from 8 leprosy-affected families as well as another case and an unrelated control, and then the follow-up SNP genotyping of the candidate variants was studied in case-control sample sets. A rare missense variant in mitochondrial ribosomal protein S5 (*MRPS5*), rs200730619 (c. 95108402T>C [p. Tyr137Cys]) was identified and validated in 369 cases and 270 controls of Chinese descent (P_adjusted_ = 0.006, odds ratio [OR] = 2.74) as a contributing factor to leprosy risk. Moreover, the mRNA level of *MRPS5* was downregulated in *M*. *leprae* sonicate-stimulated peripheral blood mononuclear cells. Our results indicated that *MRPS5* may be involved in leprosy pathogenesis. Further studies are needed to determine if defective *MRPS5* could lead to impairment of energy metabolism of host immune cells, which could further cause defect in clearing *M*. *leprae* and increase susceptibility to infection.

## Author summary

Leprosy is an infectious disease caused by *Mycobacterium leprae*, and the global new case detection rate remains high after the introduction of multidrug therapy for almost four decades with a significant reduction in prevalence. The identification of possible new genetic loci involved in susceptibility to leprosy is needed to the further understanding of the pathogenesis of leprosy and help achieve final elimination of leprosy. Here, we report a potential leprosy-associated variant in mitochondrial ribosomal protein S5 (*MRPS5)* by using whole exome sequencing performed on 28 individuals including 8 leprosy-affected families and the follow-up SNP genotyping of the candidate variant in case-control sample sets. We provide genetic evidence and expression data suggesting that *MRPS5* is a novel leprosy-associated gene which may play an important role in the pathogenesis of leprosy.

## Introduction

Leprosy is a chronic infectious disease caused by *Mycobacterium leprae (M*. *leprae)* and mainly affects the skin and the peripheral nerves, leading to disfigurement and permanent disability if patients were not treated promptly and properly [1]. Since the introduction of multidrug therapy according to the recommendations of the World Health Organization more than three decades ago, leprosy control and the outlook for patients have been significantly improved, however, the global new case detection rate remains high, which has plateaued at about 210,000 new cases annually (http://www.who.int/lep/epidemiology/en/). Although in-depth studies have been conducted, it remains unclear how the patients contract leprosy and which factors determine severity and progression of this disease. It is reported that only about 5 percent of the people exposed to *M*. *leprae* would develop leprosy in endemic countries [2] with a broad spectrum of clinical disease [3], especially in developing disfigurement and disability [4,5]. In view of the low-frequency variability of *M*. *leprae* [6], the difference of individual responses to *M*. *leprae* suggests that the host genetic background may play an important role in the occurrence and development of leprosy [7,8]. It has been demonstrated that the strongest risk factor for developing this disease in close contacts of leprosy patients is genetic relatedness [9]. The twin studies indicated the increased concordance rates of disease per se and its clinical forms among monozygotic compared with dizygotic twins [10,11]. Significant efforts have been made to identify leprosy susceptibility genes. Scientists in Canada have identified *PARK*2 and *PACRG* genes as major leprosy susceptibility genes for the first time in Vietnamese and Brazilian populations [12,13], but no association was found between *PARK2*/*PACRG* polymorphisms and susceptibility to leprosy in the Han Chinese population [14,15]. So far, dozens of variants associated with leprosy have been identified through Genome-wide association studies (GWAS) and candidate-gene studies in the Chinese population, such as those in *CCDC122*, *LACC1* (previously *C13orf31*), *NOD2*, *TNFSF15*, *HLA-DR-HL-DQ*, *RIPK2*, *IL23R*, *RAB32*, *BCL10*, *IL18RAP*/*IL18R1*, and *IL12B* [16–22].

Although GWASs have been successful in identifying genetic variants associated with complex diseases, GWAS does have important limitations. First, the effect sizes of risk variants identified by GWAS are generally small (odds ratios < 1.5), which may have limited clinical value, but nevertheless could help understand the underlying biological mechanisms of disease. Secondly, relying on association studies based on Linkage disequilibrium, GWAS might not be well suited to detect rare mutations having large effects on disease risk. And for most diseases, the identified susceptibility loci explain only a small part of heritability of complex diseases, even in diseases where very large sample sizes have been analyzed, for instance, leprosy. The genome-wide significant susceptibility loci for leprosy identified by GWAS could explain only 13.53% of phenotypic variance [21]. Finally, the disease-associated loci identified by GWAS are mainly located in non-coding regions of the genome, which could be difficult to narrow down causal genes and carry out functional experiments. Therefore, additional mutations with greater effects on the disease need to be identified to solve the so-called "missing heritability" problem [23,24]. The human genome contains approximately 180,000 exons (protein-coding regions of a gene), which are collectively called exome. An exome is about 30 mega base pairs in length and contains about 85% of known disease-related variants [25]. In 2009, exome sequencing has been successfully applied to identify the candidate genes of a rare dominantly inherited disorder for the first time [26], and then extended to study on other complex common diseases [27,28].

We first reported three new common genetic susceptible loci, one in *GAL3ST4* and two in *CHGB* identified by whole exome sequencing (WES) on four leprosy patients and four healthy relatives from two leprosy families in Henan and Yunnan Province [29]. In this study, we intended to identify novel and rare protein-coding variants through bigger-scale WES with more patients. We found that a rare missense variant in mitochondrial ribosomal protein S5 (*MRPS5*) (MIM: 611972) contributes to leprosy susceptibility in Chinese patients.

## Materials and methods

### Ethics statement

This study was approved by the Medical Ethics Committee of Beijing Friendship Hospital, Capital Medical University (approval number 2018-P2-123-01) and was carried out in accordance with The Code of Ethics of the World Medical Association (Declaration of Helsinki), and written informed consent was obtained from each of the participants at the time of sample collection. For participants who were under 18 years of age, written informed consent was obtained from a parent/guardian.

### Subjects

Participants included 377 leprosy cases (108 females) and 271 controls (140 females) (Table 1). Firstly, twenty-eight individuals including 26 persons from eight leprosy families and two sporadic individuals were selected for WES based on the number of affected individuals along with DNA concentration and purity of blood samples. In the eight selected families, there was at least one leprosy patient per family. Moreover, at least both parents and one child in each family had eligible DNA available for WES sequencing. We sequenced at least one affected and one unaffected individual per family. Altogether, we performed WES on 15 individuals affected by different spectrum of leprosy composed of 2 Lepromatous leprosy (LL), 9 Borderline lepromatous leprosy (BL), 3 Borderline tuberculoid leprosy (BT) and 1 Tuberculoid leprosy (TT), as well as 13 unaffected individuals. Rare variants (minor allele frequency (MAF) lower than 1% in the 1,000 Genomes Project data [30]) present in at least one patient but absent in control(s) within more than one family while absent in all the unaffected individuals, with a MAF lower than or equal to 0.5% in the Exome Aggregation Consortium (ExAC) Browser (version ExAC. r0.3.1) [31] were primarily identified for validation and further verification.

**Table 1:** Clinical and demographic information of individuals with leprosy and control individuals from China.

	WES	Replication
**Individuals with leprosy**		
Total number	15	55 *+307
Age range in years (mean ± SD)	21–83 (46.3 ± 17.4)	13–91 (45.6 ± 15.8) ^a^
Number of females	4 (26.7%)	104 (28.7%)
**Control individuals**		
Total number	13	258
Age range in years (mean ± SD)	23–97 (57.0 ± 19.2) ^b^	8–87 (34.8 ± 18.5) ^c^
Number of females	6 (46.2%)	134 (51.9%)

WES, whole-exome sequencing.

^a^ Fifteen individuals with leprosy had missing information regarding age.

^b^ One control individual had missing information regarding age.

^c^ Eighteen control individuals had missing information regarding age.

* 55 cases were used to reduce the list of candidate variants, and the variants with a frequency > 2/55 were ultimately selected to validate in 307 leprosy cases and 258 controls.

**Fig 1:**
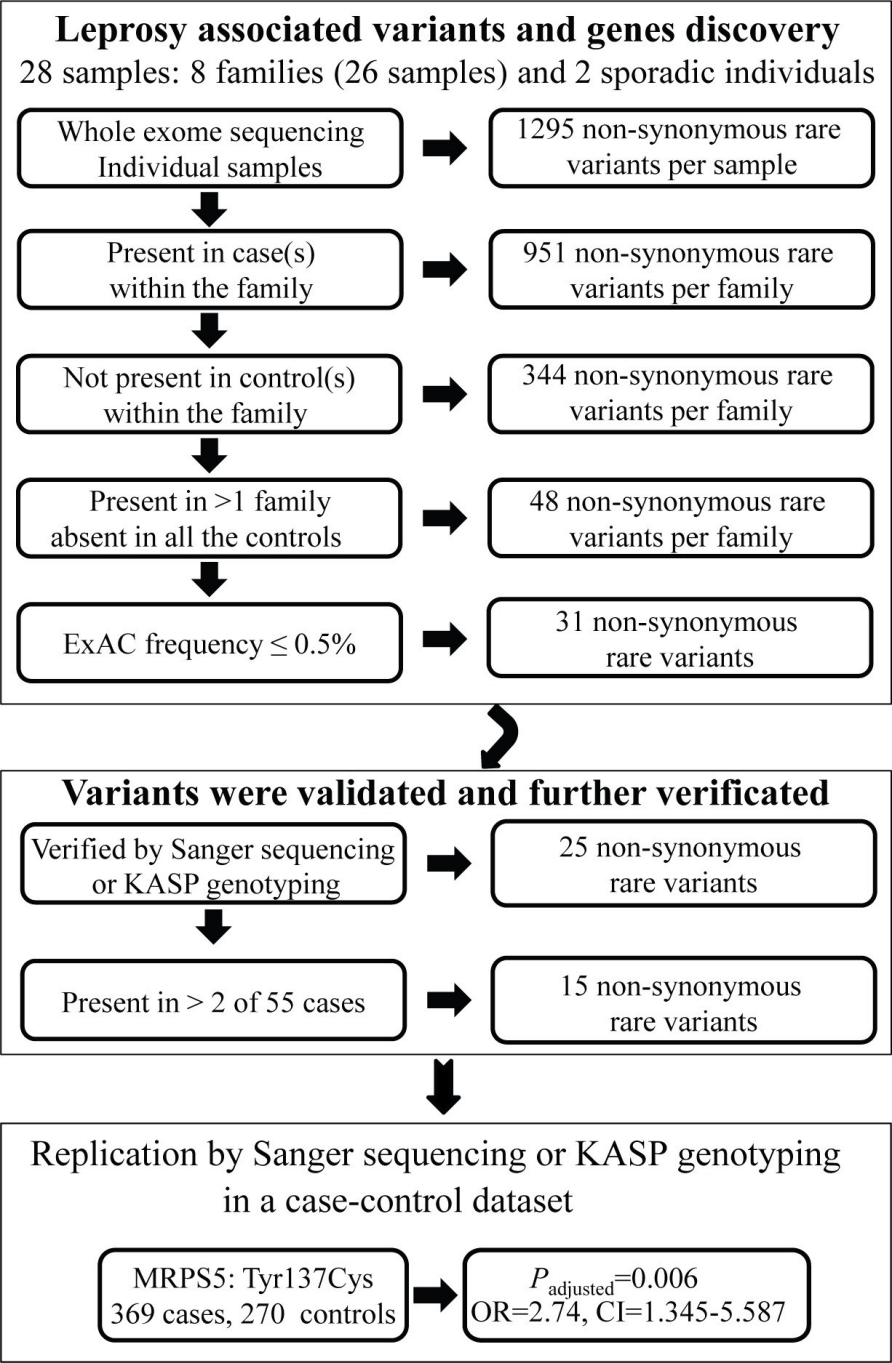
The analytical framework for the identification of leprosy susceptibility genes. The diagram shows the processes used to filter the variants identified by whole exome sequencing, which led to the identification of the mitochondrial ribosomal protein S5 (*MRPS5*) (*Y137C*) variant. The diagram also shows the subsequent genetic analyses in case-control data sets that validated the association of the *MRPS5* Tyr137Cys variant with risk for leprosy. ExAC, Exome Variant Server; KASP, KBioscience Competitive Allele-Specific Polymerase chain reaction; CI, confidence interval; OR, odds ratio.

Secondly, the preliminary selected mutations were validated by Sanger sequencing or the KBioscience Competitive Allele-Specific Polymerase chain reaction (KASP) assay. To further narrow down the number of candidate variants, we performed Sanger sequencing and KASP genotyping on 55 patients with leprosy. The rare variants with a frequency more than two in the 55 patients were ultimately selected to validate in 307 leprosy cases and 258 controls (Fig 1). However, some of the samples were not successfully amplified and/or sequenced due to their low quantity or poor quality. All cases of leprosy were diagnosed based on clinical presentations as well as slit-skin smear examination, and were classified into the spectrum of leprosy according to Ridley-Jopling criteria. Some of these patients have been described in our previous molecular epidemiological studies [32,33]. Healthy control subjects are the healthy relatives of these patients or living in or near the endemic regions [34,35]. The data of East Asians (EAS) from 1000 Genomes Project data [30] were acquired as the general control population.

### Whole exome sequencing and SNP genotyping

Genomic DNA was extracted from whole blood, formalin-fixed paraffin-embedded or ethanol-fixed skin biopsy specimens with DNeasy Blood & Tissue Kit (Qiagen, Hilden, Germany). Coding exons and their flanking DNA regions were captured via AgilentSureSelect Human All Exon V6 kits System/NimbleGen SeqCap EZ Exome v3+UTR(96M) (Agilent Technologies, Inc., Santa Clara, CA, USA) followed by a paired-end (PE150) high-throughput sequencing using Illumina HiSeq X10 platform (Illumine Inc., San Diego, CA, USA). Raw sequence reads were aligned to the reference human reference genome hg19 using Novoalign (Novocraft Technologies). Base and SNP calling was performed with SNP SAMtools [36], and Genome Analysis Toolkit (GATK) [37] software and ANNOVAR [38] was used for SNP functional annotation and filtering. On average, 95% of the exome had no less than 100× coverage. SNPs identified with a quality score lower than 19 and read depth of coverage lower than 5 were removed. The transition/transversion ratio is around 3.0 for SNPs inside exons and about 2.2 elsewhere (S1 Table). This step was performed by the Annoroad Gene Technology Co. Ltd. We identified all rare variants shared by affected individuals within at least two families and absent in all the unaffected ones. Variants with a frequency ≤ 0.5% in the ExAC were selected. The selected variants were then validated by Sanger sequencing or KASP genotyping. SNPs were genotyped using Sanger sequencing based on the use of specific primers in S2 Table or KASP genotyping technologies. The KASP genotyping was performed using Fluorescent Resonance Energy Transfer at Shanghai Baygene Biotechnology Co. Ltd.

### mRNA expression analysis

A differential expression analysis of mRNA was conducted to investigate the transcription alterations of target genes during *M*. *leprae* infection using the available data of Gene Expression Omnibus (GEO). Two datasets were downloaded and reanalyzed: (1) dataset GEO: GSE100853, a genome-wide search for expression quantitative trait loci before and after stimulation with sonicate of *M*. *leprae* in whole-blood from 51 unrelated individuals from Vietnam with borderline leprosy [39]; (2) dataset GEO: GSE74481, which included the mRNA profiles in leprosy skin lesions of 24 individuals with multibacillary leprosy including 10 BB, 10 BL, and 4 LL, 20 individuals with paucibacillary leprosy consisting of 10 TT and 10 BT, 14 individuals with type I reaction, and 9 individuals with type II reaction, and normal skin biopsies of nine healthy individuals used as controls [40].

Human peripheral blood was collected from thirty-nine subjects with wild-type *MPRS5* through venipuncture using sterile vacutainer tubes containing the anticoagulant ethylenediaminetetraacetic acid (EDTA) (BD Biosciences), and then peripheral blood mononuclear cells (PBMCs) were isolated via density gradient centrifugation with Ficoll-Paque Plus (GE Healthcare). PBMCs (2×10^6^ cells/ml) were incubated in 24-well cell culture plates for 24 hours at 37°C with 5% CO_2_ in RPMI 1640 with or without *M*. *leprae* sonicate (2 mg/ml, NR-19329, NIH, USA; final concentration 10μg/ml). Total RNA was then extracted from PBMCs through phenol-chloroform method using Trizol (Ambion) reagent following the manufacturer’s protocol. Extracted RNA was treated with DNase1 to remove any potential DNA contamination. cDNAs were synthesized from the total RNA, using the GoScript Reverse Transcription System (Promega). The expression levels of target genes were analyzed by real-time PCR using an Applied Biosystems 7500 Fast Real-Time PCR System. Reactions were performed with the PowerUp SYBR Green Master Mix (ABI). The primers used are listed in S2 Table. The Ct values of the target gene were normalized using β-actin gene to obtain ΔCt. The variance in the ΔCt values between the *M*. *leprae* sonicate-stimulated and unstimulated PBMCs was calculated to obtain ΔΔCt. The fold change of the target gene was calculated by the 2^-ΔΔCt^ method [41].

### Statistical analysis

Hardy-Weinberg equilibrium in founders was tested using an exact test as implemented in PLINK software package version 1.07 [42] (http://pngu.mgh.harvard.edu/purcell/plink) and was met for the selected SNPs (S3 Table). Association of leprosy affection status was calculated using Transmission Disequilibrium Test (TDT) as implemented in PLINK. Gene-based burden test were performed by using the Sequence kernel association test (SKAT) R-package [43,44]. In silico prediction of the functional effect of the identified variants was conducted by using SIFT (http://sift.jcvi.org/www/SIFT_BLink_submit.html) and Polyphen (http://genetics.bwh.harvard.edu/pph2/) algorithms. Using Pearson Chi-square test or with Yates’ Continuity Correction for the single-locus association analysis, the allele frequencies of the missense variants were compared between the leprosy and control individuals. Logistic regression analysis with gender as the covariate was used to generate the adjusted P value (P_adjusted_). The SPSS statistical software package ver.20.0 (SPSS Inc., Chicago, USA) was used for the statistical analysis. P values less than 0.05 were considered statistically significant. The sample size of the replication stage were computed with Quanto software (v.1.2.4) [45] using the following parameters: MAF = 0.05, the prevalence of the disease = 0.0001, and significance level = 0.05 (two sided). At least 257 pairs of case and control samples were needed to obtain an OR of 2.0 with a desired power of 80% under an additive model [28].

## Results

### Demographic characteristics of the eight Chinese families with leprosy and whole exome sequencing of leprosy families

**Fig 2:**
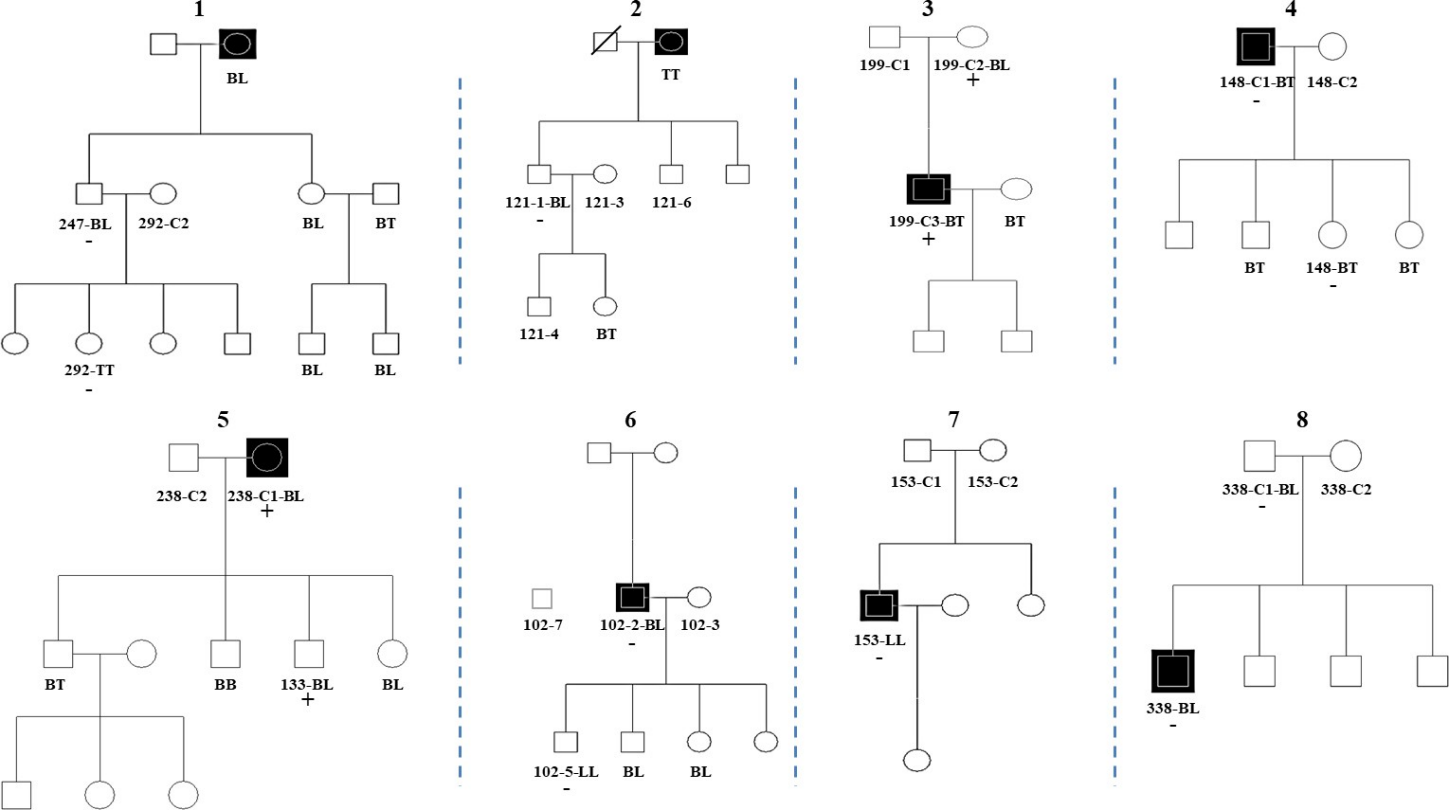
The eight families in which the candidate variants were initially found. The sequenced patients are labeled with specimen numbers and clinical forms, simultaneously unsequenced patients are labeled only with clinical forms, while the unaffected individuals are labeled only with specimen numbers. C indicates contact. TT: Tuberculoid leprosy, BT: Borderline tuberculoid leprosy, BL: Borderline lepromatous leprosy, LL: Lepromatous leprosy. Black squares indicate index cases labeled with clinical forms in the families. The diagonal line indicates the deceased individual. The “+” symbol indicates the patients positive for the Y137C variant. The “-” symbol indicates the patients negative for the Y137C variant.

We have collected a number of leprosy families to perform WES for leprosy susceptibility loci. Among these families, trio-based WES was conducted on the high quality genomic DNA from whole blood samples of eight families with at least one case of leprosy per family. The genograms of the families are shown in Fig 2, and 14 affected and 12 unaffected members of the families used in WES were marked with the number of samples and/or type of leprosy. The demography and/or clinical features of the family members as well as two sporadic individuals are summarized in S4 Table. In the 8 families, six of them originated from Qiubei County, Yunnan Province, and the other two came from Xingyi City, Guizhou Province. Among the eight parent-offspring trios comprised of 24 individuals, 2, 266, 510 SNPs and INDELs were identified of which 499, 501 variants were located in exonic or splice site regions. The TDT was used to evaluate allele transmission from parents to offspring in the 8 leprosy-affected families. Subsequently, 231,176 SNPs were originally obtained and several novel common variants (MAF≥5% in the 1,000 Genomes Project data) were recognized including two novel mutations (rs16991480 and rs76791154) in CHGB [29] (S5 Table), but no rare risk variants significantly associated with leprosy were identified. Moreover, we performed gene-based burden test in the WES samples. However, no rare variants were detected to be significantly associated with leprosy, which might be due to the low depth of coverage in each gene/region or a large amount of pathogenic variants that were not being captured as qualifying variants (such as noncoding variants or more common variants filtered out by the thresholds). Therefore, another selection criterion was used to detect the rare risk variants, that is, all rare variants shared by affected individuals in at least two families but absent in all the unaffected ones, with ExAC MAF ≤ 0.5% were primarily selected. In total, 48, 117 of the 499, 501 variants (9.63%) were identified as rare variants (a frequency less than or equal to 1% in the 1000 Genomes global population). After synonymous variants had been filtered and excluded, 32,047 non-synonymous variants were found. On average, more than 1,335 rare variants within the coding regions were identified per individual within the 8 family trios (S6 Table). According to this criterion, we confirmed 48 rare coding variants shared by cases from more than one family, but absent in all the controls (S7 Table). Furthermore, 31 variants with a MAF ≤ 0.5% on the ExAC were determined for preliminary validation by Sanger sequencing (6 variants) or KASP genotyping (25 variants). And in the end, we validated 80.6% (25/31) of the selected variants. These variants were further validated by Sanger sequencing (5 variants, *MRPS5* rs200730619, *KRT39* rs17843022, *SMARCA4* rs533671711, *NSMAF* rs142542935 and *NSMAF* rs140438385) or KASP genotyping (20 variants) in a subgroup of 55 patients with leprosy to further narrow down the number of candidate variants.

### Validation of leprosy-associated nonsynonymous variants by Sanger sequencing or KASP genotyping

We performed Sanger sequencing for *MRPS5* rs200730619, *KRT39* rs17843022, *SMARCA4* rs533671711, *NSMAF* rs142542935 and *NSMAF* rs140438385 and KASP genotyping for *XRRA1* rs180810179, *HIPK4* rs192823203, *TACC2* rs140280635, *FSIP2* rs181404625, *FSIP2* rs188160736, *PRRC2B* rs201654931, *SELO* rs202018920, *FRMD8* rs117981233, *KLHL33* rs12587478, *ADCY7* rs139279676, *LRIG1* rs749143043, *TEKT1* rs771448702, *KLHL35* rs774288909, *WAC* rs2232793, *ECE2* rs373062910, *CCBL2* rs117578041, *KIAA1551* rs529008468, *PPP2R3A* rs141649812, *DCTPP1* rs143856356 and *TAOK2* rs150712108 in 55 cases of leprosy. Among these validated 25 variants, 15 variants with a frequency more than 3.64% (2/55), which consisted of *MRPS5* rs200730619, *XRRA1* rs180810179, *KRT39* rs17843022, *HIPK4* rs192823203, *TACC2* rs140280635, *FSIP2* rs181404625, *FSIP2* rs188160736, *PRRC2B* rs201654931, *SELO* rs202018920, *FRMD8* rs117981233, *KLHL33* rs12587478, *ADCY7* rs139279676, *LRIG1* rs749143043, *TEKT1* rs771448702 and *KLHL35* rs774288909, were eventually determined for validation in additional 307 leprosy cases and 258 controls (S3 and S8 Tables). In the end, the association between *MRPS5* rs200730619 and leprosy was well validated in 369 cases (36/333, carrier frequency 9.76%) and 270 controls (11/259, carrier frequency 4.07%) (P_adjusted_ = 0.006, OR = 2.74, CI = 1.345–5.587, Table 2) after removing the unsuccessfully sequenced samples. In the 36 cases carrying the *MRPS5* mutant allele, more than one third of them (14 cases) were female, and nine cases came from four different multicase families containing at least two *MRPS5* mutation carriers. While among the 11 controls harboring the mutant allele, five were female, and about two-thirds of them (seven) came from six leprosy-affected families. Among the 34 individuals with leprosy having information regarding Ridley-Jopling classification, three were diagnosed as LL, 17 were BL, one was BB, 10 were BT and three were TT. However, there was no significant association between *MRPS5* rs200730619 and any leprosy subtype.

**Table 2:** Association between leprosy and *MRPS5* (Y137C) variant.

Stage	Sample Size		Allele Count (Alternative/ Reference)		P ^a^	P_adjusted_^b^	OR ^b^	95% CI ^b^
	Leprosy cases	Controls	Leprosy cases	Controls				
*MRPS5* rs200730619: c. 95108402T>C [p. Tyr137Cys]								
WES	15	13	4/26	0/26	-	-	-	-
Reference control (1000g2016apr-EAS)				8/1000	^c^ 4.63×10^−8^	-	-	-
Replication	354	257	32/676	11/503	0.026	0.021	2.35	1.140–4.843
Combined	369	270	36/702	11/529	0.008	0.006	2.74	1.345–5.587
Combined (All individuals)			36/702	19/1529	1.00×10^−7^	-	-	-

The table shows the allele count for leprosy patients and healthy controls. WES, samples were analyzed by whole-exome sequencing; replication, the samples were used for replication; 1000g2016apr-EAS, East Asian population data from the Genomes Project version 2015 August data [30] was used as the general control population. a. The P values were calculated by Pearson's Chi-squared test or with Yates’ Continuity Correction. b. The P values, ORs and 95% confidence intervals (CIs) were adjusted for gender. c. The P value were calculated by Pearson's Chi-squared test with Yates’ Continuity Correction.

### Biological involvement of *MRPS5* in leprosy

**Fig 3:**
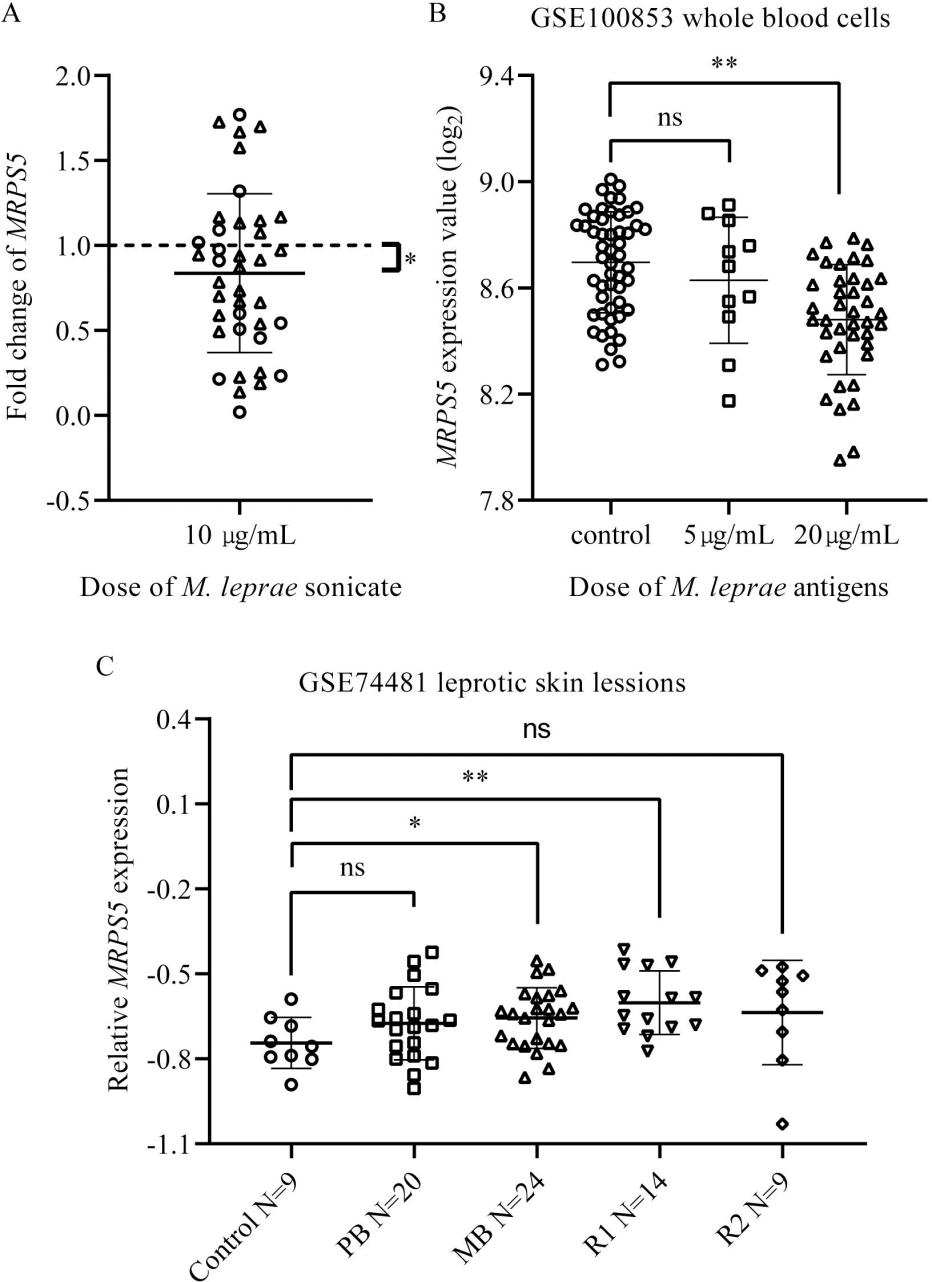
Variation of *MRPS5* mRNA expression levels in PBMCs stimulated by *M*. *leprae* antigens and in leprotic skin biopsies. (A) Downregulation of mRNA expression levels of *MRPS5* in peripheral blood mononuclear cells of individuals with or without leprosy stimulated by *M*. *leprae* sonicate. N = 39, *: *P* <0.05. Each hollow cycle represents an individual with leprosy (n = 13), each triangle represents an individual without leprosy (n = 26). The dotted line at 1 represents the fold change of unstimulated PBMCs. *Y* axis represents fold change vs unstimulated PBMCs. P value is calculated using the Paired Samples *t* Test. (B) Differentially expressed *MRPS5* in whole-blood from individuals with borderline leprosy after stimulation with *M*. *leprae* sonicate. The expression dataset GEO: GSE100853 [39], which consists of 51 unrelated Vietnamese leprosy patients, was used to determine gene expression. Whole blood cells from each subject were divided into two equal parts, one was stimulated with *M*. *leprae* sonicate (5 μg/mL for 11 subjects and 20 μg/mL for 40 subjects), while the other remained untreated was used as control. **: P <0.01, ns: not significant. (C) *MRPS5* mRNA expression in leprotic skin biopsies. The analysis was performed on the basis of the microarray expression data GEO: GSE74481 [40]. The dataset contains samples from leprotic lesions of 24 multibacillary (MB) leprosy patients, 20 paucibacillary (PB) leprosy patients, 14 patients with type I reaction (R1), and nine patients with type II reaction (R2), and meanwhile normal skin biopsies from nine healthy subjects were used as controls. *: P <0.05, **: P <0.01, ns: not significant.

An evolutionary comparison of MRPS5 p. Tyr137Cys showed that residue Tyr137 is highly conserved across different vertebrate species (S1 Fig). The variant, rs200730619, which is restricted to humans, has not been reported in ClinVar (http://www.ncbi.nlm.nih.gov/clinvar/). It has been reported that MRPS5 and the MRP protein family are the main actors in metabolic lifespan regulation [46]. Akbergenov et al. demonstrated that the human *MRPS5* V336Y mutation confers mitoribosomal misreading [47]. Therefore, rs200730619 may be a potential loss-of-function variant, and the mutant *MRPS5* might have decreased activity during physiological processes against infection. We evaluated the alterations in *MRPS5* mRNA expression in PBMCs from 39 wild-type individuals, which included 13 cases (4 LL, 7 BL and 2 BT) and 26 controls in response to treatment with *M*. *leprae* antigens, and found downregulated expression of *MRPS5* mRNA after stimulation with *M*. *leprae* sonicate (P = 0.036; Fig 3A).

In the meantime, we estimated the variations in *MRPS5* mRNA expression during *M*. *leprae* infection based on dataset GEO: GSE100853 [39]. With increased dosage of *M*. *leprae* antigens (20 μg/mL), the *MRPS5* mRNA expression level was significantly decreased (p = 8.71×10^−9^; Fig 3B). However, we observed an inverse expression pattern of the *MRPS5* mRNA level in dataset GEO: GSE74481 [40], that is, the *MRPS5* mRNA levels in leprotic skin biopsies of multibacillary individuals were significantly higher than those in control individuals (P = 0.036; Fig 3C). Moreover, mRNA expression of *MRPS5* was much higher in the skin lesions of individuals with type I reaction (P = 0.004; Fig 3C) than that in healthy individuals on the basis of dataset GEO: GSE74481 [40].

## Discussion

Over the last decade, a significant number of studies using next-generation sequencing have identified various susceptibility genes and variants associated with leprosy [8,13,21,22,28,29,48,49]. In the present study, WES was performed in 8 families with at least one case of leprosy, and 31 variants shared by affected individuals within at least two families and absent in all the unaffected ones with a MAF ≤ 0.5% in the ExAC were selected for further confirmation. Then the interest genes of potential rare protein-coding variants contributing to leprosy susceptibility were identified in a number of cases and controls. Finally we were able to identify the missense variant rs200730619 (c. 95108402T>C [p. Tyr137Cys]) within the *MRPS5* gene associated with leprosy (P_adjusted_ = 0.006, odds ratio [OR] = 2.74), which was included in the SNPs originally obtained using TDT. The association was also present when we used the 1000 Genomes Project data-EAS dataset [30] as the general control population (Table 2). There was a statistically significant difference in the frequency of Tyr137Cys in *MRPS5* between familial cases and sporadic cases (15/95 in familial versus 21/274 in sporadic cases, P = 0.02). In the meanwhile, *MRPS5* gene expression was significantly decreased in response to stimulation of *M*. *leprae* sonicate (Fig 3A), which is consistent with the reported dataset GEO: GSE100853 [39] (Fig 3B). However, *MRPS5* mRNA levels were significantly higher in individuals with multibacillary leprosy as well as type I reaction compared to controls in the light of dataset GEO: GSE74481 [40] (Fig 3C). The inconsistencies between GSE100853 and GSE74481, that is blood-based and tissue-based results may be due to differences in sample types [50], sample sizes and the different technology platforms. Although rs200730619 is not expected to be protein damaging by SIFT and Polyphen, and may not cause the disease, it may worsen the outcome, which is supported by the result that mRNA expression of *MRPS5* was much higher in the skin lesions of individuals with type I reaction than that in healthy individuals on the basis of dataset GEO: GSE74481.

The leprosy-associated variant rs200730619 (c. 95108402T>C [p. Tyr137Cys]) is located in exon of *MRPS5* in chromosomal region 2q11.1; and its MAF is highest in East Asian populations, and much lower in other populations from the 1000 Genomes Project dataset [30]. With the help of the ExAC Browser [31], the largest exome database to date, we observed a similar allele frequency pattern of rs200730619: 6.136e-03 in 8, 638 East Asians, 1,211e-04 in 16, 512 South Asians, 8.658e-05 in 11, 550 Latinos, and this allele is absent in 10,234 Africans; 73, 134 Europeans and 906 others. However, why this leprosy risk allele of *MRPS5* is specifically enriched in East Asian populations and how it contributes to the population risk for leprosy remain to be determined. As yet no studies have reported an association between rs200730619 and disease risk. It has been suggested that mitochondrial ribosomal proteins (MRPs) could be central to the effectiveness of HIV infection [51], and expression of MRPs was increased in dengue shock syndrome patients [52]. Moreover, several MRPs were involved in inflammatory and oxidative responses induced by *Candida albicans* [53]. *MRPS5* is one of the MRPs that have the strongest correlation with oxidative phosphorylation, and uncoupling of mitochondrial oxidative phosphorylation through uncoupling proteins strongly reduces mitochondrial reactive oxygen species (ROS) emission [54]. In the meantime, MRPS5 deficiency significantly reduced ROS production and nuclear translocation of *MRPS5* promoted increased expression of glycolytic proteins and transformation of metabolic pathways [55]. It has been reported that *M*. *leprae* viability is partially dependent on both host cell metabolic activities, and *M*. *leprae* could drastically reduce host cell mitochondrial membrane potential and avoid ROS generation [56]. Thus, dysregulation or dysfunction of *MRPS5* might exert influence on mitochondrial metabolism in host responses to *M*. *leprae* infection. It is conceivable that defective *MRPS5* could lead to impaired energy production in host immune cells, which could induce defect in killing or clearing *M*. *leprae* from the host. Further studies are needed to validate this in *MRPS5* knockdown immune cells in the future.

During the course of our work, another research group reported exome sequencing in Han Chinese individuals affected by leprosy. They found that a rare missense variant in *HIF1A* [MIM: 603348] and a common missense variant in *LACC1* [MIM: 613409] contribute to leprosy susceptibility in Han Chinese [28]. We checked the two risk variants in *HIF1A* and *LACC1*, but found no association between *LACC1* or *HIF1A* variants and leprosy susceptibility in our samples (S3 Table). The main reasons for this might be the relatively small sample size of our case-control study and the kinship among some of the cases and controls and the low frequency of the variant *HIF1A* rs142179458 (MAF = 0.001971 in ExAC dataset). We identified several families carrying the *MRPS5* Tyr137Cys variant and this variant was enriched in leprosy families compared to sporadic leprosy cases which demonstrate the power of using multiplex leprosy families for variant discovery.

The present study has some potential limitations. First, the number of novel identified risk mutations relies on the discovery sample sizes. In this study, we sequenced only 28 individuals in the WES discovery stage, there must have been additional rare risk mutations missed. When the previously reported WES data of four leprosy patients and four healthy relatives from two leprosy families in Henan and Yunnan Province were included to screen candidate variants [29], three novel rare nonsynonymous variants which are only present in cases from at least two families but absent in all the controls, i.e. rs746426634 in *STYK1* (MIM: 611433), rs75765336 in *GBGT1* (MIM: 606074) and rs141225250 in *GCC2* (MIM: 612711) were confirmed by Sanger sequencing and further determined in the 55 leprosy cases. At last, the minor allele of rs75765336 in *GBGT1* whose carrier frequency was greater than 3.64% (2/55) was subjected to further verification, and there was no statistically significant difference in the carrier frequency of *GBGT1* mutation between the cases and the controls (21/255 vs. 14/217, P = 0.493). Future studies with bigger sample size will provide novel insights into genetic susceptibility to leprosy. Second, 31 non-synonymous variants were selected based on the allele frequency, which may miss some significant variants, so all the rare variants (MAF<1% in the 1,000 Genomes Project data) present in case(s) but absent in control(s) within each family have been listed in S9 Table to benefit the field. Third, further validation of our current results in combination with other candidate genes including the recently reported ones [28] in different populations is needed to illuminate the relative importance of these variants in the pathogenesis of leprosy.

In summary, we have uncovered a missense variant in *MRPS5* that contributes to leprosy risk in Chinese patients. We have provided genetic evidence to indicate *MRPS5* as a susceptibility locus for leprosy. Further studies are needed to validate the association in more independent populations and to functionally characterize the role of *MRPS5* in potentiating the susceptibility to leprosy.

## Supporting information

S1 Fig **Sanger sequencing of subjects harboring genotypes TT and TC of rs200730619 (A) and protein sequence alignments showing the conservation of Tyr137 in 9 vertebrate species (B).** The protein sequences were retrieved from NCBI (https://blast.ncbi.nlm.nih.gov/Blast.cgi). The sequence ID number is given after the species name.
(TIF)

S1 Table The transition/transversion ratios of SNPs in genome and exonic regions identified through whole exome sequencing. TS: transition; TV: transversion. The numbers 1–8 indicate the 8 families.
(XLSX)

S2 Table Sanger sequencing primer pairs used for validation and gene expression of variants identified by whole exome sequencing. (XLSX)

S3 Table Genotype counts and Hardy-Weinberg test statistics for the selected SNPs. CHR: Chromosome; SNP: SNP identifier; TEST: Code indicating sample; A1: Minor allele code; A2: Major allele code; GENO: Genotype counts; O (HET): Observed heterozygosity; E (HET): Expected heterozygosity; P: H-W p-value; AFF: cases; UNAFF: controls; CHISQ: chi-square (χ2) value; P ^a^: p-value in a chi square test; P_adjusted_, OR, 95% CI: adjusted p-value, odds ratio and 95% confidence intervals in a logistic regression analysis with gender as the covariate.
(XLSX)

S4 Table Demographic and clinical data for 15 leprosy patients and 13 healthy controls subjected to WES. UN: unknown. YNQB: Qiubei county, Yunnan Province. GZXY: Xiyi county, Guizhou Province. The patient numbers are shown in bold font. The numbers 1–8 indicate the 8 families. a A sporadic patient, b A sporadic control.
(XLSX)

S5 Table Novel common variants suggested by transmission disequilibrium test, a family-based association study on 8 leprosy families. CHR, Chromosome; SNP, SNP identifier; A1, Minor allele code; A2, Major allele code; T, Transmitted minor allele count; U, Untransmitted allele count; OR, TDT odds ratio; CHISQ, TDT chi-square statistic; P, TDT asymptotic p-value; A:U_PAR, Parental discordance counts; CHISQ_PAR, Parental discordance statistic; P_PAR, Parental discordance asymptotic p-value; CHISQ_COM, Combined test statistic; P_COM, Combined test asymptotic p-value.
(XLSX)

S6 Table Demographic features and summarized results of the exome-sequenced samples within 8 leprosy families. The patient numbers are shown in bold font. The individuals not belonging to the 8 family trios are in italics.
(XLSX)

S7 Table Forty-eight rare coding variants shared by affected individuals from at least two families but absent in all the unaffected ones. The rare variants validated by Sanger sequencing are shown in bold font. The variants excluded by Sanger sequencing or KASP genotyping are underlined. cytoBand: Chromosome Band; AA change: amino acid change; Alt: Alternative; Ref: Reference; +: alt; -: ref; 1000g 2016apr_all: 1000 Genomes Project version 2015 August data; ExAC_all: Exome Aggregation Consortium Browser v3.1; SIFT score predicts whether an amino acid substitution affects protein function; PolyPhen-2: Polymorphism Phenotyping v2, predicts possible impact of an amino acid substitution on the structure and function of a human protein; SNV: single nucleotide variants.
(XLSX)

S8 Table Genotyping results of the 17 variants in the case-control study. NS: not successful; ND: not done. The upper numbers indicate the families some cases and controls came from. The two variants in *HIF1A* and *LACC1* were also included.
(XLSX)

S9 Table The rare variants present in case(s) but absent in control(s) within each family are listed. (XLSX)
