# Supplementary figures and images for: Polymorphisms in mitochondrial ribosomal protein S5 (MRPS5) are associated with leprosy risk in Chinese
Source: PLoS Negl Trop Dis. 2020 Dec 23;14(12):e0008883. doi: 10.1371/journal.pntd.0008883 (PMC7757804; doi:10.1371/journal.pntd.0008883)

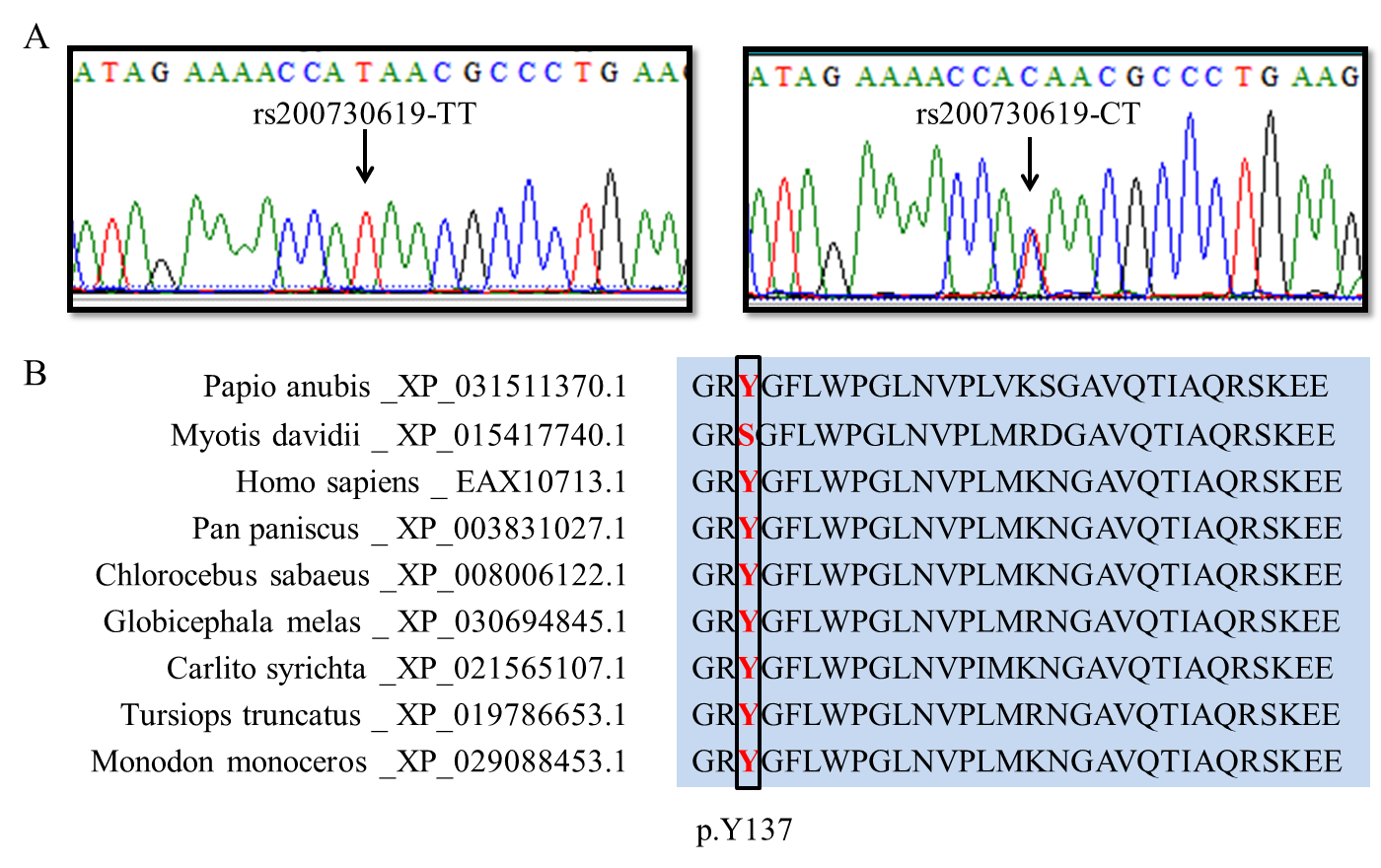

Supplement: S1 Fig — Sanger sequencing of subjects harboring genotypes TT and TC of rs200730619 (A) and protein sequence alignments showing the conservation of Tyr137 in 9 vertebrate species (B). The protein sequences were retrieved from NCBI (https://blast.ncbi.nlm.nih.gov/Blast.cgi). The sequence ID number is given after the species name. (TIF) [file pntd.0008883.s001.tif]
